# Editorial: The use of chemotherapy in treating gastric cancers

**DOI:** 10.3389/fonc.2022.974023

**Published:** 2022-07-26

**Authors:** Lei Huang, Yan Shi

**Affiliations:** ^1^ Department of Oncology, Ruijin Hospital, Shanghai Jiao Tong University School of Medicine, Shanghai, China; ^2^ Medical Center on Aging of Ruijin Hospital, Medical Center on Aging of Ruijin Hospital (MCARJH), Shanghai Jiao Tong University School of Medicine, Shanghai, China

**Keywords:** gastric cancer, chemotherapy, predictive markers, safety and efficacy, precision and targeted oncology

Despite a declining trend in incidence globally, gastric cancer (GC) ranks 5^th^ in incidence and 4^th^ in mortality among 36 cancers across 185 countries. In 2020, over 1,089,000 new cases of GC and approximately 769,000 GC-associated new deaths were estimated worldwide (1–4). A noticeable proportion of GC cases are diagnosed at later stages, which can rule out resection as a primary treatment option, leaving systemic options which commonly involve chemotherapy, either as monotherapy or combined with other therapies (5–8). Chemotherapy is also frequently utilized in the adjuvant and/or neoadjuvant setting for cases manageable with resection (9–11). Notably, chemotherapy may be prone to causing adverse effects in places beyond the malignant sites (12).

This Research Topic, which has collected six high-quality original researches, aimed to highlight some of the novel emerging advancements pertaining to chemotherapy for GC, as well as the ways that physician scientists help patients with GC receiving chemotherapy by streamlining the treatment, and by delivering the drugs *via* individualized routes.

Adjuvant chemotherapy may need to be administered timely in patients with resected GC upon recovery of performance statuses (9); Chen et al. explored the risk factors and prognostic impacts of delayed (>60 days after resection) or omitted adjuvant chemotherapy, which may be unacceptable, in resected TNM stage II-III GC by retrospectively analyzing data of 1520 patients undergoing radial gastrectomy, and demonstrated that delayed or omitted postoperative chemotherapy was significantly associated with inferior overall survival (OS) and disease-free survival (DFS). Various factors associated with delayed or omitted adjuvant chemotherapy were further revealed, including female sex, old age, history of intraabdominal surgery, serious postoperative complications, etc. These data call for further standardization of GC care.

Notably, not every patients with resected non-metastatic GC can benefit from adjuvant chemotherapy, and it is important to precisely identify and screen the subgroup of patients for whom chemotherapy-associated benefits truly outweigh harms, to avoid overtreatment (12–16). Identification of novel indicators for chemotherapy sensitivity remains an unmet need. Perineural invasion (PNI) is not rare in GC; Tao et al. analyzed the associations between PNI and efficacy of fluoropyrimidine (FU)-based adjuvant chemotherapy in patients with radically resected stage IB-III GC from two independent retrospective cohorts. Both univariable and multivariable analyses showed that adjuvant chemotherapy was significantly associated with both enhanced OS and DFS only in PNI-positive cases irrespective of cancer stages, but not in PNI-negative ones. For the underlying mechanisms, the authors (13) found that PNI-positive GCs had significantly lower expressions of biomarkers associated with hypoxia and resistance. Thus, PNI may assist with prediction of adjuvant chemotherapy benefits in resected GC; however, the findings should be further validated in prospective cohorts.

Adverse events especially severe ones should be carefully watched out for patients with GC receiving chemotherapy (4), among which neutropenia is a common one; Xiao et al. investigated the associations between preoperative nutritional status and Grade ≥3 neutropenia during the first cycle in patients with GC receiving adjuvant oxaliplatin plus capecitabine chemotherapy based on retrospective data from a single tertiary referral hospital, and found that several nutritional parameters including serum pre-albumin level, prognostic nutritional index, and pre-cycle neutrophil count were significant independent risk factors. The findings may call for the need of prophylactic granulocyte colony-stimulating factor use in selected patients at a high risk of severe neutropenia during chemotherapy. Notably, the fact that neutrophils can promote GC progression should also be cautiously factored into treatment decisions (17).

In patients with resectable advanced GC undergoing resection, adverse events associated with neoadjuvant chemotherapy may have an impact on postsurgical complications; during exploration of the safety of neoadjuvant chemotherapy for GC, Wu et al. aimed to clarify if neoadjuvant oxaliplatin plus S-1 (SOX) chemotherapy and the related adverse events were associated with the risk of postsurgical complications. The authors found that preoperative comorbidities, clinical T4b stage, and more cycles (5-6 vs 3-4) of preoperative chemotherapy were independent risk factors for more frequent postoperative complications, while the presence of preoperative SOX chemotherapy, neoadjuvant chemotherapy-associated adverse events, or their severity was not significantly associated with the occurrence of postsurgical complications.

For metastatic GC, chemotherapy may be the most important treatment modality, and innovative regimens with both good efficacy and safety profiles are desperately needed to enhance prognosis (8). Feldbrügge et al. for the first time retrospectively evaluated the safety of systemic chemotherapy (with or without the VEGFR2 antagonist ramucirumab) combined with pressurized intraperitoneal aerosol chemotherapy (PIPAC), a local chemotherapy method using the laparoscopic technique, for GC with peritoneal metastasis. Ramucirumab may cause wound healing problems, while the authors found that the addition of ramucirumab to systemic chemotherapy before PIPAC did not increase the risk of postoperative adverse events, regardless of the length of the treatment-free interval before PIPAC (even with an interval as short as 2 weeks before PIPAC), and was thus a safe alternative for the management of GC with peritoneal metastasis (16). Importantly, randomized controlled trials remain urgently needed to further verify the efficacy of PIPAC management and to ascertain the optimal combination and timing of inductive and intermittent systemic therapy.

After removal of esophageal cancer, neoplastic gastroesophageal anastomotic stricture (NGAS) is a highly clinically challenging condition. Xie et al. retrospectively analyzed 50 patients with NGAS receiving arterial infusion chemotherapy (AIC), and demonstrated the safety, feasibility, and efficacy of AIC for NGAS after esophagectomy. Further prospective and randomized evidence is needed to confirm the findings.

Together, this Research Topic has included interesting and important publications (13–18) addressing the attractive and intriguing topic: “The Use of Chemotherapy in Treating Gastric Cancers” (**Figure 1**), and covered the following contents: The prognostic impacts of delayed and omitted adjuvant chemotherapy and the associated factors, perineural invasion as a predictive marker for benefits from adjuvant chemotherapy and the underlying mechanisms, nutritional factors associated with neutropenia during the first cycle of adjuvant chemotherapy, associations of neoadjuvant chemotherapy and related adverse events with postoperative complications, safety of pressurized intraperitoneal aerosol chemotherapy together with ramucirumab-included chemotherapy for GC with peritoneal metastasis, and arterial infusion chemotherapy for neoplastic gastroesophageal anastomotic stricture after esophagectomy. Further research is warranted to identify more targeted novel chemotherapeutic options, and to refine those already existing with the goals of higher efficacy and lower toxicity (14, 16–18).

**Figure 1:**
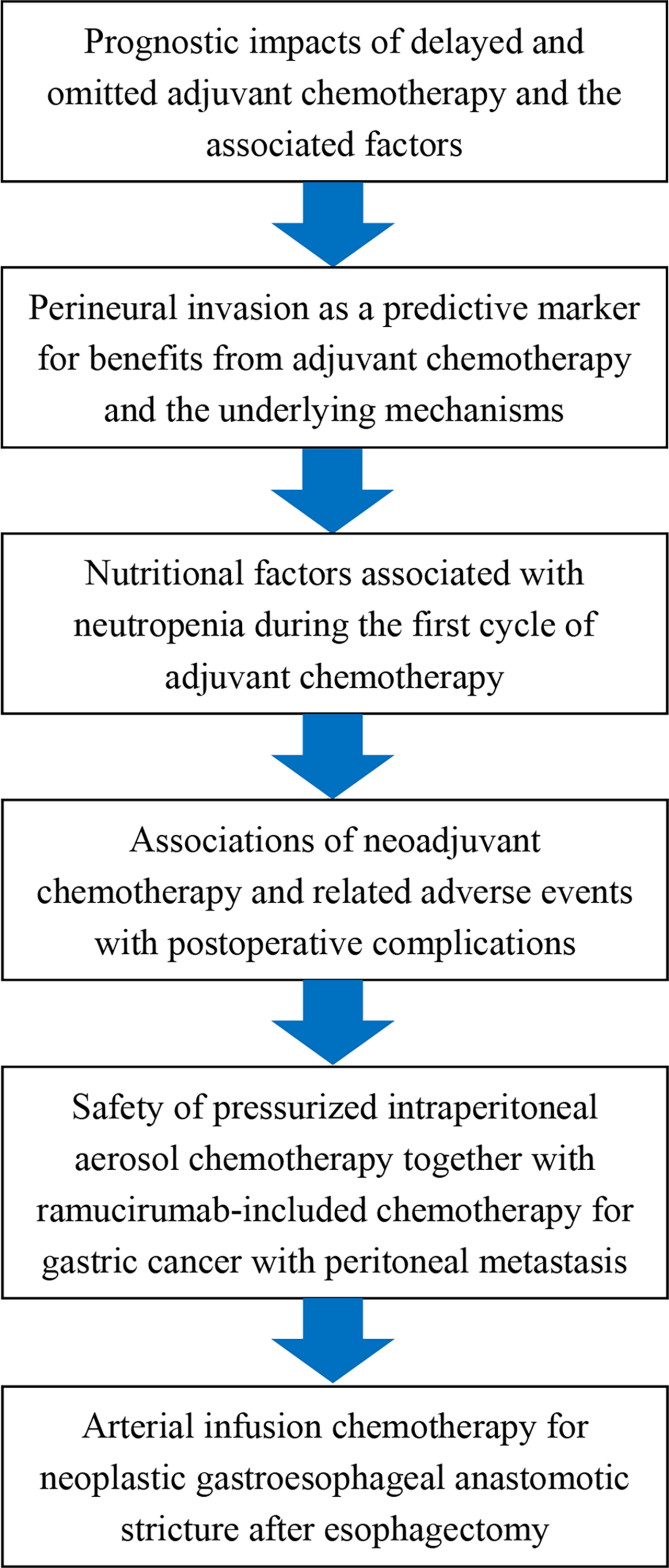
Contents of the research topic: “The Use of Chemotherapy in Treating Gastric Cancers”.

In this era of precision and targeted medicine, future researches may need to focus more on forefront aspects including the application of advanced techniques using artificial intelligence for more personalized prediction of net benefits and noteworthy side effects associated with chemotherapy, novel treatment modalities including immune cell therapies (e.g., CAR-T and CAR-NK therapies) and treatment based on innovative antibody drug conjugates (ADCs, like GQ1001 and DS-8201) (19), utilization of digital mobile medicine interventions (e.g., applets) to improve treatment compliance and case surveillance in patients with GC receiving chemotherapy, etc.

## Author contributions

Conception or design: LH and YS. Acquisition, analysis, or interpretation of data: LH and YS. Drafting of the manuscript: LH. Critical revision of the manuscript for important intellectual content: LH and YS. Administrative, technical, or material support: LH and YS. Both authors have approved the current version of the manuscript for submission and publication.

## Funding

Our study was supported by Shanghai Pujiang Program (21PJ1409700), and the Start-up Fund for the Introduction of High Level Talents by Ruijin Hospital, Shanghai Jiao Tong University School of Medicine. The funders had no role in study design; in the collection, analysis, or interpretation of data; in the writing of the report; or in the decision to submit the paper for publication.

## Conflict of interest

The authors declare that the research was conducted in the absence of any commercial or financial relationships that could be construed as a potential conflict of interest.

## Publisher’s note

All claims expressed in this article are solely those of the authors and do not necessarily represent those of their affiliated organizations, or those of the publisher, the editors and the reviewers. Any product that may be evaluated in this article, or claim that may be made by its manufacturer, is not guaranteed or endorsed by the publisher.

## Abbreviations

GC, gastric cancer; OS, overall survival; DFS, disease-free survival; PNI, perineural invasion; SOX, oxaliplatin plus S-1; PIPAC, pressurized intraperitoneal aerosol chemotherapy; NGAS, neoplastic gastroesophageal anastomotic stricture; AIC, arterial infusion chemotherapy; ADC, antibody drug conjugate.
